# The impact of lockdown policy on depressive symptoms among pregnant women in China: mediating effects of internet use and family support

**DOI:** 10.1186/s41256-021-00193-4

**Published:** 2021-03-26

**Authors:** Yongjie Zhou, Ruoxi Wang, Lei Liu, Ting Ding, Lijuan Huo, Ling Qi, Jie Xiong, Jie Yan, Lingyun Zeng, Jiezhi Yang, Suyi Song, Gaolanxin Dai

**Affiliations:** 1 Shenzhen Kanning Hospital, Shenzhen, 518020 China; 2 School of Medicine and Health Management, Tongji Medical College, Huazhong University of Science and Technology, Wuhan, 430030 Hubei China; 3 Research Center for Rural Health Services, Hubei Province Key Research Institute of Humanities and Social Sciences, Wuhan, 430030 Hubei China; 4 Qingdao Mental Health Center, Qingdao University, Qingdao, 266034 China; 5 Department of Psychiatry, Affiliated Brain Hospital of Guangzhou Medical University (Guangzhou Huiai Hospital), Guangzhou, 510000 China; 6 The First School of Clinical Medicine, Southern Medical University, Guangzhou, 510000 China; 7 School of Health Science and Nursing, Wuhan Polytechnic University, Wuhan, 430023 China; 8 ESSCA School of Management, 1 Rue Joseph Lakanal – BP 40348, 49003 Cedex 01 Angers, France; 9 Grenoble Ecole de Management, 12 Rue Pierre Semard, 38000 Grenoble, France; 10 Shenzhen Health Development Research Center, Shenzhen, 518028 China

**Keywords:** Pregnant women, Maternal depressive symptoms, Lockdown, COVID-19, Internet use, Family support

## Abstract

**Background:**

Although more and more attention has been paid to the psychological consequences of the lockdown policy amongst pregnant women, the underlying mechanism linking the lockdown policy to maternal depression has not been studied in the context of China. This study aimed to explore the association between the lockdown policy and maternal depressive symptoms, and whether such association was mediated by internet use and/or family support.

**Methods:**

This cross-sectional study used multi-stage sampling techniques in central and western China. Data were collected from 1266 pregnant women using a structtured questionnaire that measured internet use, family support, and depressive symptoms. The Patient Health Questionnaire-9 (PHQ-9) was used to measure depressive symptoms. Internet use was measured by length of usage and varierity of purpose for internet use. Family support was measureed by spousal support and parental support. The structural equation modelling was employed to conduct mediation analysis to test the specificity of the hypothetical paths.

**Results:**

Overall, 527 respondents (41.63%) presented depressive symptoms. The lockdown policy was negatively associated with depressive symptoms in pregnant women (β = − 0.925, 95% CI = −1.510, − 0.360). The impact of the lockdown policy on depressive symptoms was partially mediated by internet use (β = 1.589, 95% CI = 0.730, 2.807) and family support (β = − 0.162, 95% CI = − 0.341, − 0.017), accounting for 42.67% of the total effect.

**Conclusions:**

The lockdown policy was generally associated with fewer depressive symptoms in pregnant women. The lockdown policy increased maternal depressive symptoms through increased internet use, but decreased maternal depressive symptoms through enhanced family support. The findings suggest that the psychological consequence of the lockdown policy may vary across different populations, and warrant the need to take into consideration the features of subgroups.

## Introduction

Depression, a type of non-communicable disease, has attracted wide attention in both developed and developing countries due to its high prevalence and heavy burden. With nearly 350 million individuals suffering from depression, it accounts for 12.7% of the all-cause mortality [1] across the globe. According to the World Health Organization (WHO), it has been ranked the third leading cause of the global burden of disease, and is expected to rank first by the year 2030 [2]. With respect to disadvantaged groups, it has been widely acknowledged that pregnant women are vulnerable to depression due to the sharp social, psychological, and hormonal changes accompanied by the major life event of pregnancy [3]. At a prevalence rate of over 10% [4, 5], pregnant women are at a significantly higher risk for depression than the general population [2]. Aside from the heavy burden to the affected individuals, maternal depression may also have profound effects on the offspring, affecting the physical, cognitive, and psychological development during childhood and adolescence [6]. In addition, maternal depression has been associated with higher risk for chronic illnesses in offspring during adulthood [7, 8]. Therefore, this calls for special attention to be paid to the psychological health of women during pregnancy.

Since December 2019, the outbreak of coronavirus disease (COVID-19) has spread across the globe rapidly and widely, and soon became a worldwide pandemic [9]. To date, more than 19 million cases of COVID-19 infections have been reported, as well as more than 0.7 million deaths [10], causing severe burden to the infected individuals, their family, and society as a whole. Since rapid human-to-human transmission has been confirmed, the Chinese government has adopted a lockdown strategy for the purpose of cutting off the routes of virus transmission. The nationwide travel restriction policy came into effect on January 25th, when the last of the 30 provinces launched the Level 1 Emergency Response [11]. Fourteen cities in Hubei Province adopted the lockdown policy by January 24th [12]. Under the lockdown policy, public transportation was suspended, public places were closed for social distancing, and residents were required to self-quarantine in their homes. The lockdown strategy was soon implemented in several countries and regions [13, 14].

As evidence increasingly suggested a positive impact of the lockdown strategy on containing the spread of COVID-19 [15, 16], an increasing number of researchers started to raise concerns on the psychological consequences of the lockdown policy in regard to the sudden changes to daily livelihood. Specifically, some researchers called for more attention to be paid to pregnant women, since there were concerns about the wellbeing of the unborn child during COVID-19. A lack of access to expected prenatal care under the lockdown policy [17, 18] may intensify psychological distress, as well as the sharp drop of social interactions due to quarantine [19] could increase the chances of developing maternal depressive symptoms.

The actual impact of the wide-range lockdown policy on depression in the current situation remains largely unknown for the following reasons. First, extant studies speculated negative psychological consequences by attributing factors such as frustration, boredom, and inadequate information as the main stressors during quarantine [20]. In fact, these speculations largely came from studies during the outbreak of Severe Acute Respiratory Syndrome (SARS) or Middle East Respiratory Syndrome (MERS) [19, 21, 22] rather than from early evidence during COVID-19 [23]. However, Alterna et al. [24] argued that the sharp change in the social context questions whether the findings from the past confinement studies still hold true in the current scenario, as one can easily preserve communication, work, and education via the widely accessible Internet nowadays. Second, limited studies that looked into the psychological consequences of the social distancing during COVID-19 yielded mixed findings [20]: some observed a significantly higher risk for depression among individuals affected by quarantine than their counterparts [25], whereas other did not [26, 27]. Further, prior literature reported large heterogeneity in terms of the reaction toward social distancing across different subgroups of the general population, revealing that younger individuals were more likely to be self-centered and therefore respond with more negative emotions [28], whereas those over 30 years of age were more family oriented and presented relatively fewer psychological symptoms [29]. Social factors such as having a student status [30], living alone, being under peer pressure, or having family conflicts tended to have increased risk for depression under the lockdown policy, whereas being married or having a cohabitant reduced such risk [31]. These findings warrant stratified analysis to further understand whether, and how, the lockdown policy has affected depression in specific subgroups.

Amongst the small number of studies that concerned the psychological consequences of the lockdown policy during COVID-19, studies that further explored the underlying paths through which the lockdown policy affected one’s mental health were scarce. The majority of limited studies regarded increased Internet use as an important mediator. Prior studies observed that policies such as lockdown and social distancing largely increased Internet use [32, 33] for purposes such as social interaction [32, 34, 35] and seeking health-related information [36, 37]. This was especially seen in the cases of individuals affected by the lockdown policy since they were restricted to home confinement, and the Internet was the only source to collect information about what is going on outside [31]. In regard to the relationship between Internet use and depression, some researchers warned that excessive Internet use, such as binge-watching television or very frequent social media use, may lead to adverse consequences, such as behavioral addition or depression [38–40]. In the specific context of COVID-19, some researchers were concerned that emotions such as fear, frustration, and low moods can spread rapidly on social media [19], and therefore, severely affect those who spend a lot of time online [28, 31]. The condition may become more worrying due to the inaccurate, or even misleading, information from the false reports [41]. Moreover, prior studies revealed that residents from the epicenters/lockdown areas are more inclined to seek, and more sensitive to, information related to epidemic situation than their counterparts from other areas [42], this is to say that it is likely that residents under lockdown restrictions have higher risk for depressive symptoms than their counterparts. In addition, information about the supply shortage posted on social media created panic buying [43], which in turn, could further heighten panic and stress among those who must rely on online shopping. Besides the theoretical deduction, one study observed a positive association between the lockdown policy, increased screen exposure, and depressive symptoms [30]. This study focused only on office workers and students, whether a similar relationship is evident in pregnant women warrants further investigation.

Compared to the concerns on the adverse psychological consequences of the lockdown policy, significantly fewer studies explored its positive effect on one’s mental health. We considered family support, a widely acknowledged protective factor for depressive symptoms under adverse conditions [44–47], in light of the theoretical relationship between the lockdown policy and family support. As Behar-Zusman et al. [48] demonstrated, family bond may be further strengthened under the lockdown policy since the home confinement provided increased opportunities for family members to share more time together, communicate with each other, support each other, and confront challenges together. The positive effect of the lockdown policy may be especially true in China’s context of a traditional male-dominant culture: men are the ones who spend significant amount of time working outside to support the family [49], and tend to have a significantly larger social network than women [50]. This often leads to a limited time for husbands to communicate with pregnant women, and therefore, resulting in pregnant women having limited family interactions with their husbands prior to the implementation of the lockdown policy. The sharp increase in family time may contribute to higher levels of satisfaction toward family relationships. In addition, it has been documented that those who experienced disasters such as the Wenchuan Earthquake [51], and attacks on the World Trade Center [52] reported increased sense of family cohesion/support, suggesting that family members tend to support each other when confronting disasters [53]. Whether the lockdown policy led to increased family support, and therefore, better emotional health in current times deserves further quantitative analysis.

**Fig. 1:**
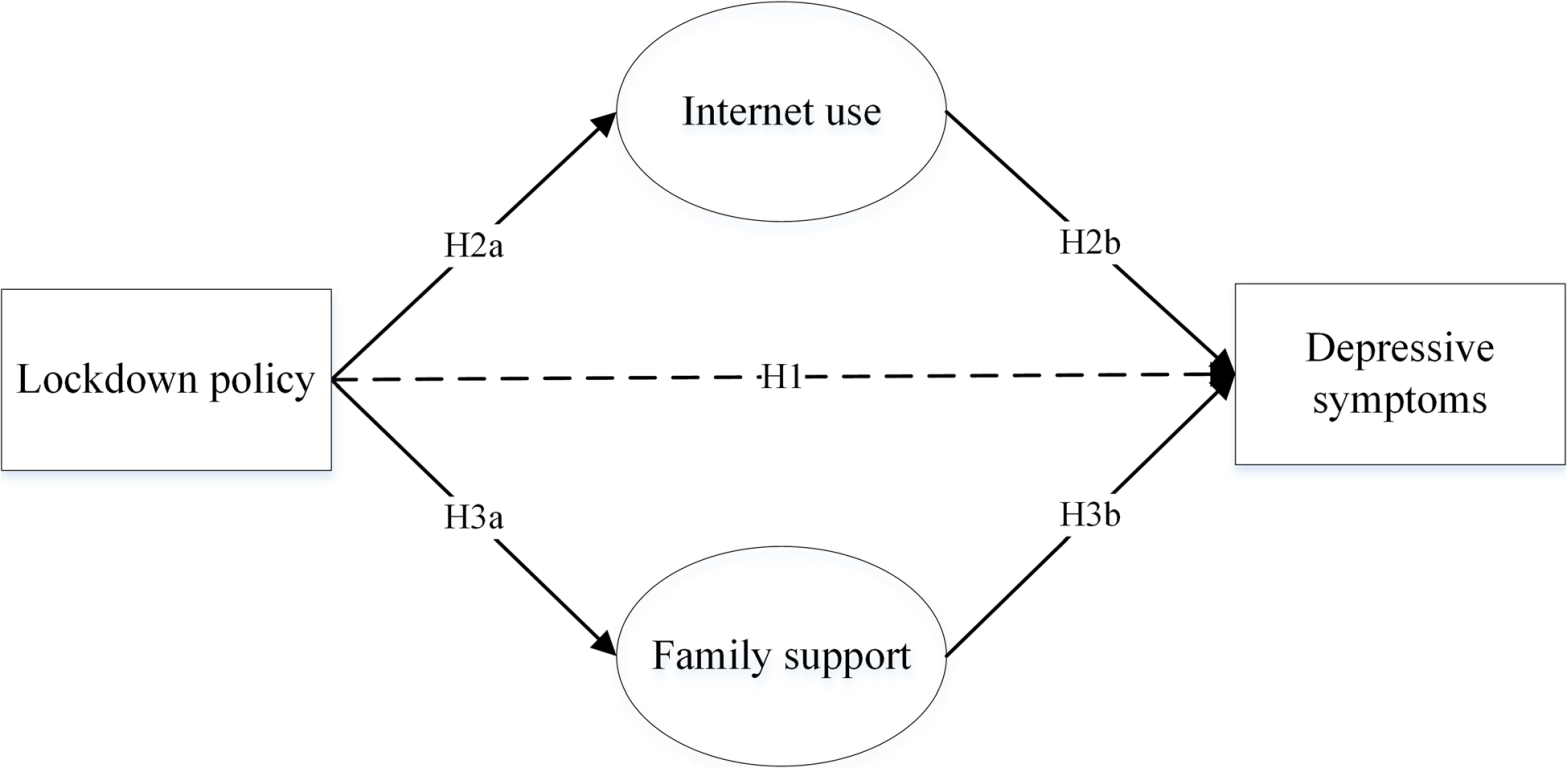
Research model and hypothesized relationships

To fill these research gaps, this study aims to understand the mechanism of the relationship between the lockdown policy and depressive symptoms among pregnant women in China. Based on existing literature, we hypothesize that in general, the lockdown policy poses a negative effect on maternal psychological health (H1); on the one hand, pregnant women affected by the lockdown policy and more Internet use (H2a) would report more depressive symptoms (H2b); on the other hand, those who were affected by the lockdown policy and experience higher level of family support (H3a), which is negatively associated with depressive symptoms, would report less depressive symptoms (H3b)) (Fig. 1).

## Methods

### Setting and study population

This cross-sectional survey employed a multi-stage sampling technique to enroll participants between March 30th to April 26th, 2020. In the first stage, we selected Wuhan City (capital of Hubei Province) and Lanzhou City (capital of Gansu Province) due to the following reasons. First, policies such as lockdown, and other types of travel restrictions, depended on the level of Public Health Emergency Response in China. As aforementioned, the nationwide travel restriction policy came into effect on January 25th, when the last of the 30 provinces launched Level 1 Emergency Response [11]. The first batch of eight provinces downgraded their emergency response level to Level 3 Emergency Response, and lifted travel restrictions by the end of February [54], including Gansu (February 21st), Liaoning (February 22nd), Guizhou (February 23rd), Yunnan (February 24th), Guangxi (February 24th), Inner-Mongolia (February 25th), Hainan (February 26th), and Shaanxi (February 28th). The announcement of Level 3 Emergency Response indicated that these areas were at low risk for COVID-19, and the local governments switched their policy priority to restoring order, production, and everyday life [55]. Gansu province was the first to switch to Level 3 Emergency Response [56], suggesting that the residents in Lanzhou City were more likely to return to normal daily life prior to the implementation of this study than their counterparts from other provinces. As of April 8th, Wuhan, the capital city of Hubei Province, was the last city to lift the full lockdown policy in China. Moreover, the city continued to be under tight control measures until May 2nd, when the government lowered its emergency response level to Level 2 [57]. Under such circumstances, participants from Wuhan City and Lanzhou City may represent pregnant women more affected and less affected by the lockdown policy, respectively. Second, Hubei Province (Central China) and Gansu Province (Western China) are not contiguous with each other, which largely rules out the potential spill-over effect of the wide lockdown measures in Hubei [58]. In the second stage, we selected a regional maternal and child health care center in each city in China’s context. The underlying reason is as follows: the professional maternal and child health care resources are mostly located in the regional centers of each city. Meanwhile, more than 95% of healthcare insurance coverage [59] largely removed the income-related inequality in the healthcare-seeking process. As a result, most of those seeking prenatal care regard regional centers as their first choice. In this case, we selected a regional maternal and child health care center in each city to collect data from dispersed populations. In the third stage, convenience sampling was employed to enroll participants from these two centers. We sent a QR code for an online questionnaire to healthcare professionals responsible for conducting maternity examinations at the sample sites. These healthcare professionals invited eligible pregnant women to participate in this study when they came in for their antenatal examinations. It has been documented that over 90% of Asian women have gestation less than 41 weeks [60], which suggests that most of the Chinese women with over 40 weeks’ gestation would be those who were hospitalized and waiting for delivery in China’s context. As this study aimed to understand the experience of pregnant women at home rather than living in a medical institution, we included women who were up to 40 weeks pregnant or less (similar inclusion criteria can be found in Özkan et al. [61]); and lived in the local community during the COVID-19 pandemic. We excluded those with a history of mental disorders (e.g. depression, anxiety, or cognitive impairment). Informed consent was obtained from all participants. Ethical approval was obtained from the Ethics Committee for Scientific Research at Institute of Psychology, Chinese Academy of Sciences.

### Variables

#### Outcome variable

We employed a Chinese version of Patient Health Questionnaire-9 (PHQ-9) to measure depressive symptoms among pregnant women. The participants were asked 9 questions subject to the 9 diagnostic criteria of the Diagnostic and Statistical Manual of Mental Disorders 5th Edition (DSM-V) for depression. For each question, the participants were asked to rate the frequency they experienced the symptom from 0 to 3, representing ‘not at all’ to ‘nearly every day’. The total score ranges from 0 to 27, with higher scores indicating more severe symptoms. This scale has been widely used across the globe [62], and shows high sensitivity and specificity in the screening of depression [63]. The Chinese version has also been proven to have high validity [64]. In the present study, the Cronbach’s alpha coefficient for the scale was 0.87. According to Kroenke K et al. [65], PHQ-9 scores of 0–4 represent absence of a depressive disorder, whereas PHQ-9 scores of 5–9, 10–14 and 15 or above represent mild, moderate, and severe depression, respectively. In this case, a score of 5 was used as the cut-off point to indicate presence of depressive symptoms following Guille et al. [63] in sample description, whereas the total score of PHQ-9 was used in the mediation analysis.

#### Main explanatory variables

Lockdown policy: as aforementioned, participants from Wuhan City were coded as ‘affected by the lockdown policy’, whereas those from Lanzhou City were coded as ‘less affected by the lockdown policy’.

Internet use: we measured this latent variable from two dimensions, including length of usage and purposes for Internet use. For the former variable, participants were asked how many hours per day they normally spent on online activities during the COVID-19 pandemic. Length of usage ranges from 0 to 24 h/day. For the latter variable, we employed a multiple-choice question “what is the main purpose of your internet use” (search for professional information/ seek pleasure or fun/ dispel loneliness such as chat with friends) following Liang et al. [66]. Taking the pregnancy nature of the participants, we added two options for the purpose following Lagan et al. [67], including ‘search for health related information’ and ‘purchase items for pregnancy’. For the aforementioned five purposes, each of the item was coded as 1 if it was chosen, otherwise, coded as 0. We counted the variety of purposes for Internet use, which ranges from 0 to 5.

Family support: as most of the pregnant women in China live with their husbands and/ or their own parents, we measured family support based on the amount of spousal support and parental support. Participants were asked ‘to what extent will your spouse try their best to help you when things go wrong’ and ‘to what extent will your parent(s) try their best to help you when things go wrong’. They were asked to rate this on a scale of 1 to 4, representing ‘none’ to ‘great’ respectively. These two items came from the Chinese version of Social Support Rating Scale (SSRS) that evaluates one’s sources of social support (including family, friends, colleagues, neighbors, employment, etc.) and their utilization of it [68]. This scale has been widely employed in China, and proven to have high reliability and validity [69].

#### Potential confounding variables

This study considered socio-economic status, pregnancy related and health behavior variables as covariates. Socio-economic status variables included age, education (≤ high school/ college/ undergraduate/ ≥ post-graduate), employment (unemployed/ employed), living arrangements (with husband only/ with parent(s) only/ with husband and parent(s)/ other), annual household income (RMB < 80,000/ 80,000-300,000/ > 300,000), and financial loss during COVID-19 (no financial loss/ < 20,000/ 20,000-49,999/ ≥50,000). We classified the respondents into inner-city and outer-city residents according to their household living regions defined by National Bureau of Statistics [70]. Pregnancy related variables included planned pregnancy (no/ yes), and first child (no/ yes). Health behavior variables included alcohol consumption (never/ ceased/ yes), and smoking (never/ ceased/ yes).

### Statistical analysis

Frequencies and percentages were calculated for categorical data, median value and interquartile range (IQR) were used to describe non-normally distributed continuous variables, and mean value and standard deviation (SD) were computed to describe normally distributed variables. Chi-square tests, Mann-Whitney tests, and t-tests were performed accordingly. A confirmatory factor analysis (CFA) with robust maximum likelihood estimation was performed to assess the goodness of fit of the measurement model regarding the two latent variables, namely Internet use and family support. With a satisfactory measurement model, a structural equation modelling (SEM) was then performed to measure the hypothesized relationships among lockdown policy, Internet use, family support, and depressive symptoms. The SEM controlled for all covariates significant in descriptive analysis and was tested by robust weighted least squares. A normed Chi-square (χ^2^/*df*), Comparative Fit Index (CFI), Root Mean Square Error of Approximation (RMSEA), and Standardized Root Mean Square Residual (SRMR) were employed to measure the goodness of fit of the model [71]. The CFA results indicated that all of the factor loadings were above 0.48 with a significance of *p* < 0.05. Meanwhile, the indicators χ^2^/*df* = 3.737 (*p* < 0.001), CFI = 0.981, RMSEA = 0.047, and SRMR = 0.029 were observed, suggesting good model fit [71]. The mediating effect was tested by bias-corrected bootstrap with 10,000 bootstrap samples. Wald Chi-square test was performed to examine the difference between direct and indirect effects. Data were analyzed using R Version 3.5.1.

## Results

### Basic characteristics of the respondents

Basic characteristics of the whole study population, as well as of those who presented low or high risk for depressive symptoms, are shown in Table 1. Of 1266 participants, a great proportion were with undergraduate or above educational attainment, living in inner city, partnered and employed. The majority of respondents did not consume alcohol or smoke, planned for pregnancy, expected to have their first child, and were affected by the lockdown policy.

**Table 1:** Basic characteristics of the sample

	Total (*n* = 1266)		Absence of depressive symptoms (*n* = 739)		Depressive symptoms (*n* = 527)		χ^2^/t	*p*
	n	%	n	%	n	%		
**Age**							1.235	0.217
Mean (SD)	29.95	9.042	30.22	11.346	29.58	3.970		
**Education**							7.338	0.062
≤ High school	263	20.774	150	20.298	113	21.442		
College	377	29.779	203	27.470	174	33.017		
Undergraduate	541	42.733	329	44.520	212	40.228		
≥ Post graduate	85	6.714	57	7.713	28	5.313		
**Residency**							0.149	0.700
Outer city	455	36.634	267	37.083	188	36.015		
Inner city	787	63.366	453	62.917	334	63.985		
**Living arrangement**							5.521	0.137
With husband only	592	46.761	330	44.655	262	49.715		
With parents only	100	7.899	54	7.307	46	8.729		
With husband & parents	553	43.681	343	46.414	210	39.848		
Other	21	1.659	12	1.624	9	1.708		
**Employment**							1.204	0.273
Housewife	474	37.441	286	38.701	188	35.674		
Employed	792	62.559	453	61.299	339	64.326		
**Planned pregnancy**							6.882	0.009
No	384	30.332	203	27.470	181	34.345		
Yes	882	69.668	536	72.530	346	65.655		
**First Child**							0.031	0.861
Yes	846	66.877	495	67.073	351	66.603		
No	419	33.123	243	32.927	176	33.397		
**Alcohol consumption**							6.344	0.042
Never	1107	87.441	660	89.310	447	84.820		
Ceased	133	10.506	68	9.202	65	12.334		
Yes	26	2.054	11	1.488	15	2.846		
**Smoke**							1.360	0.507
Never	1210	95.577	710	96.076	500	94.877		
Ceased	51	4.028	27	3.654	24	4.554		
Yes	5	0.395	2	0.271	3	0.569		
**Annual household income (RMB)**							12.634	0.002
< 80,000	491	38.784	259	35.047	232	44.023		
80,000-300,000	690	54.502	421	56.969	269	51.044		
> 300,000	85	6.714	59	7.984	26	4.934		
**Financial loss in COVID-19 (RMB)**							17.198	0.001
No financial loss	224	18.391	149	20.927	75	14.822		
< 20,000	148	12.151	97	13.624	51	10.079		
20,000-49,999	426	34.975	220	30.899	206	40.711		
≥ 50,000	420	34.483	246	34.551	174	34.387		
**Lockdown policy**							18.352	< 0.001
Less affected	491	38.784	250	33.829	241	45.731		
Affected	775	61.216	489	66.171	286	54.269		

A total of 527 respondents (41.63%) presented depressive symptoms. Generally speaking, those who attained less education, had an unplanned pregnancy, ever consumed alcohol, had lower household income, suffered from greater financial loss, or were less affected by the lockdown policy had higher risk for depressive symptoms than their counterparts.

Table 2 depicts the Internet use behaviors, perceived family support, and depressive symptoms in different subgroups. Compared to their counterparts, those who were affected by the lockdown policy reported significantly longer duration and more purposes in the use of Internet, higher level of spousal and parental support, and less depressive symptoms.

**Table 2:** Internet use, family support and depressive symptoms in different subgroups

	Total (n = 1266)		Less affected by lockdown policy (*n* = 491)		Affected by lockdown policy (*n* = 775)		Statistics	*p*
	Median	IQR	Median	IQR	Median	IQR		
**Internet use**								
Length per day	5	(3, 6)	4	(2, 3)	5	(3, 8)	−7.627^a^	< 0.001
Diversity of purpose	3	(1, 4)	2	(1, 3)	3	(2, 4)	−10.168^a^	< 0.001
**Family support**								
Spouse support (mean, SD)	3.806	0.564	3.729	0.698	3.854	0.453	−3.867^b^	< 0.001
Parental support (mean, SD)	3.807	0.557	3.756	0.665	3.840	0.474	−2.631^b^	0.009
**Depressive symptoms**	4	(1, 7)	4	(1, 8)	3	(1, 6)	3.791^a^	< 0.001

N.B. *IQR* Interquartile range; ^a^: Mann-Whitney test, ^b^: t test

### Mediating effect of internet use & family support

**Fig. 2:**
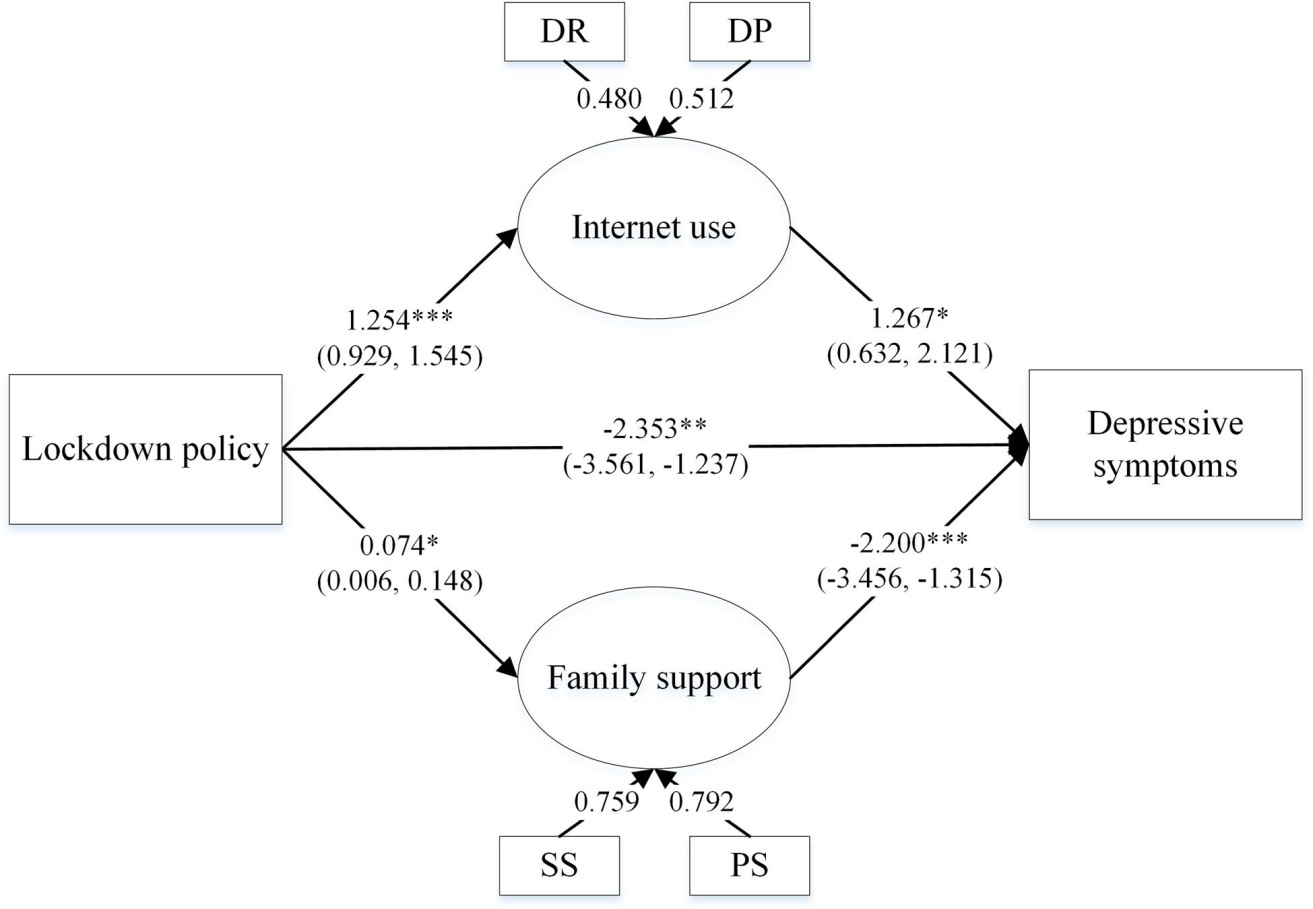
Results of the hypothesized model. N.B. DR: Duration of the Internet use, DP: Diversity of Internet use purposes, SS: Spousal support, PS: Parental support. All models controlled for age, education, living arrangement, planned pregnancy, alcohol consumption, annual household income and financial loss due to COVID-19 pandemic. *: *p* < 0.05, **: *p* < 0.01, ***: *p* < 0.001

The results of the hypothesized mediation analysis are presented in Fig. 2. The lockdown policy was negatively associated with depressive symptoms (β = − 0.925, 95% CI: − 1.510, − 0.360). The association was partially mediated by increased Internet use (β = 1.589, 95% CI: 0.730, 2.807) and family support (β = − 0.162, 95% CI: − 0.341, − 0.017). Internet use and family support contributed to an indirect effect of 42.67% on the relationship between the lockdown policy and depressive symptoms in pregnant women (Table 3).

**Table 3:** coefficients of mediation analysis

Pathway	std. Estimate	z	SE	95% CI^1^
Total effect	−0.925**	−3.130	0.296	−1.510, −0.360
Direct effect	−2.353**	−3.161	0.744	−3.561, −1.237
Indirect effect				
LD → IU → DS (2)	1.589*	2.225	0.714	0.730, 2.807
LD → FS → DS (3)	−0.162*	−1.995	0.081	−0.341, −0.017
Dominant Pathway Test				
(2) + (3)	1.427*	2.015	0.708	0.562, 2.584
(2)–(3)	1.751*	2.400	0.729	0.861, 2.994

N.B. (2): Lockdown policy → internet use → depressive symptoms; (3): Lockdown policy → family support → depressive symptoms; ^1^: 95% CI was calculated using bias-corrected bootstrap with 10000 bootstrap samples; *: *p* < 0.05, **: *p* < 0.01

## Discussion

To the best of our knowledge, the present study is the first one to examine the relationship between the lockdown policy and depressive symptoms among pregnant women in China. Meanwhile, this study is one of the very few studies that further explored the underlying mechanism of how the lockdown policy influenced depressive symptoms. The findings revealed that: 1) a significant proportion of pregnant women presented depressive symptoms; 2) with respect to pregnant women, the lockdown policy was associated with fewer depressive symptoms in China; 3) on the one hand, the lockdown policy was associated with increased Internet use, which was positively related to depressive symptoms; on the other hand, the policy was also associated with increased family support, which was related to fewer depressive symptoms.

In line with prior literature [72], this study found that over 40% of the participants presented depressive symptoms. The figure is explicitly larger compared to estimates of Chinese pregnant women prior to COVID-19 [73]. Meanwhile, the prevalence is higher than the estimates among the general public during COVID-19 [26, 27]. Echoing with extant findings [17, 30, 74], this finding indicates that pregnant women are more vulnerable to emotional problems during the COVID-19 pandemic, and therefore, deserve special attention and targeted countermeasures.

We were surprised to observe a negative association between the lockdown policy and depressive symptoms, as the finding is counterintuitive and contradictory to the findings retrieved from some studies [20, 25]. With the underlying reasons not fully comprehended, we speculate that the comparative advantage perceived from social comparison, the sense of security resulted from the reduced risk for infection, and the accompanying psychological support measures may help pregnant women feel less stressed and less depressed under the lockdown policy. Regarding social comparison, it is widely acknowledged that the degree of satisfaction/happiness one perceives does not depend on the absolute level of value, but rather, on the difference with the reference [75]. In other words, the stress and depressed mood pregnant women feel may be determined not only by the self-comparison on the before- and after- COVID-19 outbreak, but also by the lateral comparison between themselves and others who are also experiencing similar problems [75]. Right after the lockdown policy was implemented, the State Department and Health Commission of China issued a national-level notification specifically towards children and pregnant women, urging local governments to pay special attention to the safety of pregnant women and their child(ren), and adjust healthcare resource allocation strategies accordingly [76]. Under such circumstances, pregnant women from Wuhan City may perceive a higher level of safety compared to their counterparts such as other women at the same age who may have to face healthcare shortage when infected. The downward comparison may result in improved psychological function under threat [77, 78]. In terms of sense of security of reduced risk for infection, prior literature demonstrated that the lockdown policy proved to be effective not only in reducing the spread of COVID-19, but also increasing the psychological distance towards COVID-19 among residents in the pandemic regions [79]. The latter contributed to improved mental health. With respect to the accompanying psychological support measures, prior studies observed that individuals affected by quarantine received significantly more psychological help from the community or government agencies than their less affected counterparts [25]. In this case, local countermeasures towards the psychological health under the lockdown policy may offset the adverse effects of social distancing. This counterintuitive finding reminds us that the psychological consequences of the lockdown policy may vary across different populations [28, 29], and warrants the need to take into consideration the features of different subgroups. Additionally, it calls for further studies to establish a comprehensive understanding of the wide impact of the lockdown policy. Meanwhile, the finding indicates that the mental health of pregnant women in low-risk areas also deserves attention, and therefore, calls for specific interventional policies when priorities have been placed on restoring order in production and life.

In regard to the pathways by which the lockdown policy influenced depressive symptoms, this study echoed prior literature by identifying the significance of increased Internet use on one’s mental health [30], and revealed that the positive association between the lockdown policy, increased Internet use, and elevated depressive symptoms also stays true in pregnant women in the context of China. The significant association between elevated depressive symptoms with increased Internet use calls for alternatives such as physical activities to distract pregnant women from excessive Internet use [80]. In the context of COVID-19, the threat from the social media-induced panic warrants closer collaboration among policymakers, health professionals, and media experts to prevent the epidemic of information [81].

As we hypothesized, this study established a quantitative relationship among the lockdown policy, family support, and depressive symptoms. This indicates that the participants who were affected by the lockdown policy and also reported significantly higher satisfaction from spousal and parental support than their counterparts exhibited fewer depressive symptoms. Thus, this study illustrated that family support was negatively associated with depressive symptoms. Similar findings were seen in prior studies against disasters such as the Wenchuan Earthquake [51] and the attacks on the World Trade Center [52]. This finding, on the one hand, indicates the potential positive impact the lockdown policy may bring to one’s mental health [48], and on the other hand, calls for creative policies (such as mass education) to help pregnant women stay emotionally connected with their loved ones [82].

The findings should be interpreted with caution due to the following limitations. First, due to the cross-sectional design, the causal paths in the model were still based on hypothetical relationships. The actual causality awaits future studies using longitudinal design. Second, As PHQ-9 is a self-reported screening tool, future studies may benefit from diagnostic instruments. Third, the study was carried out in relatively stable stage of the domestic pandemic, which may result in an underestimation of the impact of the lockdown policy on maternal depressive symptoms. Fourth, the lockdown policy was issued as a countermeasure of the COVID-19 pandemic in China. In other words, we were unable to find a perfect control group experiencing a severe local COVID-19 epidemic, but without the lockdown policy. In this case, the effect of the lockdown policy on one’s psychological health may be compounded with that from the COVID-19 pandemic. Moreover, we selected the pregnant women from Wuhan City to measure the effect of the lockdown policy. As Wuhan City has been under lockdown for more than 2 months, the results may not be generalizable to those who were affected by shorter lockdown. Fifth, due to the limited time for survey under the pandemic, we did not collect information regarding pregnancy related complications. Future studies may yield more acute results by controlling variables such as placental or fetal presentation issues in the regression analysis.

## Conclusions

By comparing Internet use behaviors, perceived family support, and depressive symptoms between participants affected and less affected by the lockdown policy, the present study explored the mechanism of the relationship between the lockdown policy and maternal depression in China. In summary, this study yielded three main findings. First, pregnant women suffered from elevated depressive symptoms during the COVID-19 pandemic, which calls for special attention from family, community, and healthcare professionals. Second, the lockdown policy was generally associated with less depressive symptoms in pregnant women. This finding highlights the fact that the psychological consequence of the lockdown policy may vary across different populations, and warrants the need to take into consideration different features of subgroups. Third, the lockdown policy affected maternal depressive symptoms not only through increased Internet use, but also via enhanced family support. Interventions such as promoting offline interaction among family members and verifying the authenticity of news before publication may be worth consideration.

## Data Availability

The data used are available and will be provided by the corresponding author if necessary.

## Abbreviations

COVID-19: Coronavirus disease 2019
SARS: Severe acute respiratory syndrome
MERS: Middle East respiratory syndrome
PHQ-9: Patient health questionnaire-9
DSM-V: Diagnostic and statistical manual of mental disorders 5th edition
SSRS: Social support rating scale
IQR: Interquartile range
SD: Standard deviation
CFA: Confirmatory factor analysis
SEM: Structural equation modelling
CFI: Comparative fit index
RMSEA: Root mean square error of approximation
SRMR: Standardized root mean square residual
DR: Duration of the internet use
DP: Diversity of internet use purposes
SS: Spousal support
PS: Parental support
CI: Confidence interval
